# Effects of Cold Plasma Treatment Conditions on the Lipid Oxidation Kinetics of Tilapia Fillets

**DOI:** 10.3390/foods12152845

**Published:** 2023-07-27

**Authors:** Chencheng Liu, Jiamei Wan, Yuanyuan Wang, Gu Chen

**Affiliations:** 1 Hainan Engineering Research Center of Aquatic Resources Efficient Utilization in South China Sea, Key Laboratory of Seafood Processing of Haikou, School of Food Science and Engineering, Hainan University, Haikou 570228, China; lcc19970921@163.com (C.L.); yuanyuanwang0224@163.com (Y.W.); chengu1618@163.com (G.C.); 2 Collaborative Innovation Center of Provincial and Ministerial Co-Construction for Marine Food Deep Processing, Dalian Polytechnic University, Dalian 116034, China

**Keywords:** cold plasma, tilapia fillets, lipid oxidation, oxidation kinetics

## Abstract

This study investigated the effects of different cold plasma treatment conditions on the lipid oxidation kinetics of tilapia fillets. The results indicated that increasing the voltage and prolonging the treatment time of cold plasma could cause an increase in the peroxide value and thiobarbituric acid-reactive substance values of the fillets. The changes in the primary and secondary oxidation rates of the lipids in the fillets under different treatment conditions were consistent with zero-order reaction kinetics. The analysis of the fitting of the Arrhenius equation showed that the effect of treatment voltage on the activation energy of lipid oxidation was higher than that of treatment time. When the voltage was higher than 64.71 kV, the activation energy of the primary oxidation of lipids was greater than that of secondary oxidation. Within 0–5 min, the activation energy of primary oxidation first increased then decreased, and was always greater than that of secondary oxidation. Therefore, the primary lipid oxidation of tilapia was more sensitive to the treatment conditions of cold plasma.

## 1. Introduction

Tilapia is rich in nutrients, such as protein, saturated fatty acids, monounsaturated fatty acids, and polyunsaturated fatty acids. It is widely distributed in Southeast Asia and can be artificially cultivated, making it an important source of protein [1]. China is among the world’s leading producers of tilapia, with a strong aquaculture capacity. So far, tilapia fillets are primarily exported in large quantities worldwide. Owing to its high nutrition content, tilapia fillet is susceptible to microbial contamination during cold chain transportation, leading to the loss of nutritional components and a decline in taste, increasing economic losses during transportation [2]. Therefore, the non-thermal sterilization and preservation of tilapia fillets have been studied in application prospects.

As a mature sterilization technology in the food industry, heat sterilization has been widely used. However, high-temperature treatment can result in protein denaturation, changing the texture and color of food [3,4]. The high heat transfer efficiency of microwaves can lead to a reduction in the content of unsaturated fatty acids and protein denaturation in fish [5]. Long-term dry sterilization under high temperature ensures the microbial safety of canned meat, but causes protein denaturation and a decrease in the water-holding capacity, resulting in changes in texture [6]. High pressure combined with heat treatment can cause phospholipid hydrolysis in pork and promote lipid oxidation synergistically [7]. Therefore, non-thermal sterilization technologies have become a research hotspot in recent decades. Cold plasma, due to its excellent sterilization effect, is a promising alternative technology to thermal sterilization.

Plasma is a highly ionized gas in an electric field, containing positive and negative ions, electrons, atoms, ultraviolet light, and other components that are electrically neutral as a whole [8]. Cold plasma contains a large amount of active free radicals, which can effectively kill microorganisms and promote the oxidation of lipids and proteins in food [9]. Choi et al. [10] treated dried Lophiomus setigerus with a dielectric barrier discharge (DBD) system and found that the degree of lipid oxidation increased by 1.5–1.6 times as the plasma treatment time extended, and that the sensory evaluation results significantly decreased (*p* < 0.05). The treatment of *Scomber scombrus* with DBD cold plasma at 70 kV for 5 min significantly increased the degree of primary lipid oxidation, while that of secondary lipid oxidation did not change significantly [11]. Similarly, cold plasma treatment accelerated the degree of protein and lipid oxidation in *Portunus armatus* [12]. Based on our previous study, cold plasma treatment increased the lipid oxidation of tilapia [13] and Trachinotus ovatus [14]. The increased oxidation effect of cold plasma is caused by many factors, especially the treatment voltage and time. However, most of the published papers have focused on the increased oxidation degree resulting from the factors and the increased oxidation mechanism, and the oxidational kinetics of lipids caused by the treatment voltage and time are not clear yet.

Fish is rich in unsaturated fatty acids, which are prone to auto-oxidation, a process that occurs in three stages: induction, propagation, and termination. The accumulation of hydroperoxides can be used to reflect the stages of lipid oxidation. Okpala et al. [15] studied the effect of ozone treatment on the lipid oxidation of shrimp and found that shrimp lipid oxidation followed first-order kinetics. Extending the treatment time would prolong the induction period, which is conducive to lipid stability. Other studies have shown that adding antioxidants to sunflower seed oil can delay the oxidation of oil caused by microwave treatment. The linear fitting of the accumulation of hydroperoxides showed that lipid oxidation followed half-order kinetics, and the addition of antioxidants delayed the rate of oxidation [16]. The oxidation kinetics of lipids from tilapia after cold plasma treatment is not clear, and few studies about the analysis of the oxidation constant and reactive energy based on the Arrhenius equation have been published.

In order to investigate the oxidative effects of cold plasma treatment with different voltages and times on lipids from tilapia fillets and the oxidational kinetic changes during treatment, the accumulation of peroxide value (POV) and thiobarbituric acid-reactive substance (TBARS) value were used to reflect the primary and secondary oxidation rates of lipids, respectively, the oxidation indicators were fitted, and function equations were established. The differences in lipid oxidation due to variations in treatment voltage and time were analyzed by comparing the function equations and the Arrhenius equation. The test results might provide theoretical support for understanding the mechanism and kinetics of lipids oxidation caused by cold plasma.

## 2. Materials and Methods

### 2.1. Materials

Fresh tilapia fillets (weight 100 ± 5 g, without skin) were purchased from Quanyi Food Co., Ltd. in Haikou, China, and immediately transported to the laboratory on ice.

### 2.2. Cold Plasma Treatment Conditions for Tilapia Fillet Samples

**Figure 1:**
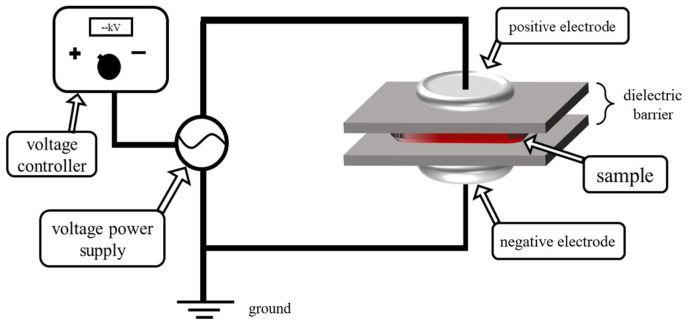
System of DBD used in this study.

The DBD cold plasma system used in this study was the same as that set up by Wang et al. [13], as shown in Figure 1. The plasma-generation device consisted of a voltage controller, a power supply (Phenix Technologies Inc., Accident, MD, USA), two aluminum electrodes, and two dielectric barrier discharges separating the upper and lower electrodes. Before plasma treatment, one fish fillet was placed in a polypropylene tray, and the box was then filled with air and sealed with a packaging machine (MAP-H360, Suzhou Senrui Fresh Equipment Co., Ltd., Suzhou, China). The sealed tray was placed between the upper and lower electrodes for direct treatment.

Treatment voltage conditions: the samples were subjected to treatment with different voltages (40, 50, 60, 70, and 80 kV) for 3 min each. Treatment time conditions: the samples were treated at 70 kV for varying times (0, 60, 120, 180, 240, and 300 s). After treatment, the samples were stored at temperatures of 4, 15, 25, 35, and 45 °C for 24 h before being analyzed.

### 2.3. Lipid Extraction

Lipid extraction was performed based on the method described by Albertos et al., with slight modifications [11]. Tilapia fillets (50 g) were ground using a meat grinder (LETGOO, Guangzhou, China) and mixed with 200 mL of a chloroform–methanol mixture (2:1, *v*/*v*) before being shaken at 37 °C for 4 h in a shaker (BSD-YX3600, Shanghai, China). The mixture was then filtered, and the filtrate was added to a KCL solution (0.88%, *w*/*v*) at a ratio of 1:2.25 (*v*/*v*). The resulting mixture was shaken for 10 min on a shaker and then allowed to stand for layer separation. The chloroform layer was collected, concentrated by rotary evaporation at 45 °C, dried with pure nitrogen, and then the fish oil was stored at −20 °C for subsequent analysis.

### 2.4. POV Determination

The method was slightly modified based on the procedure described by Wang et al. [17]. Fish oil (1–2 g) was mixed with 15 mL of a chloroform–acetic acid mixture (2:3, *v*/*v*), and then 0.5 mL of a saturated potassium iodide solution was added. The resulting mixture was shaken for 30 s and allowed to stand for 3 min in the dark at room temperature. Afterward, the mixture was removed and added to 30 mL of distilled water. The iodine liberated was titrated with standard sodium thiosulfate solution (0.002 mol/L) until the solution turned pale yellow. Next, 0.5 mL of a 1% (*w*/*v*) starch indicator was added, and titration was continued until the blue color of the solution disappeared, indicating the endpoint of the titration. In the blank group, 1 mL of distilled water was used instead of fish oil. The POV was calculated according to Equation (1), and the number of millimoles of active oxygen per kilogram of the sample was used to represent the peroxide value.

(1) $$x=\frac{(a\;-a0)\times c}{2-m}\times 1000$$

*x*: peroxide value (mmol/kg); *a*: volume (mL) of sodium thiosulfate solution consumed by the sample; *a*0: volume (mL) of sodium thiosulfate solution consumed by the blank; *c*: concentration (mol/L) of standard sodium thiosulfate solution; *m*: sample mass (g)

### 2.5. Determination of TBARS Content

Tilapia fillets (5 g) were ground and mixed with 25 mL of 7.5% (*w*/*v*) trichloroacetic acid (containing 1% EDTA). The mixture was homogenized at 6000 r/min (FJ300-SH, Shanghai, China) in a thermostatic water bath with shaking for 30 min. Subsequently, the mixture was centrifuged at 5000× *g* for 10 min, and the supernatant was filtered through Whatman filter paper. Five milliliters of the filtrate were mixed with five milliliters of 0.02 mol/L 2-thiobarbituric acid solution and placed in boiling water for 40 min, then cooled at room temperature for 1 h. The absorbance at 532 nm was measured using a UV spectrophotometer with 7.5% trichloroacetic acid and 0.02 mol/L 2-thiobarbituric acid solution mixed in equal volumes as the blank control. The TBARS value (mg/kg^−1^) was calculated from the standard curve of malondialdehyde.

### 2.6. Analysis of Lipid Oxidation Kinetics

Studies have shown that food quality changes follow zero-order kinetics during storage [18]. In this study, the zero-order kinetic model was used to reflect the primary and secondary oxidation of fish lipids induced by increased plasma treatment voltage or time [19]. The zero-order reaction equation is:

(2) $$\frac{\mathrm{dC}}{\mathrm{dt}}=\mathrm{kC}$$

where *C* represents the measurement value of lipid oxidation; *t* represents the treatment time or voltage; *k* represents the reaction rate constant; and n represents the reaction order. If the reaction follows zero-order kinetics, then *n* = 0.

Temperature can significantly affect the stability of lipids, and the Arrhenius equation effectively reflects the influence of temperature on food quality. The equation is as follows:

(3) $$k=k0\times e^{\frac{-\mathrm{Ea}}{\mathrm{RT}}}$$

*k* represents the reaction rate constant; *k*0 is the pre-exponential factor; *Ea* is the minimum activation energy required for the reaction (J/mol); *R* is the molar constant of gas (8.314 J/(mol·K)); and *T* is the absolute temperature (in K).

The impact of treatment factors on lipids can be assessed by determining the reaction rate constant using the zero-order reaction equation. The POV and TBARS values obtained from experiments were converted to logarithmic values, and the storage temperature was converted to its reciprocal. By substituting these values into Equation (3), *Ea* was obtained. The change in *Ea* was then analyzed to determine the impact of treatment factors on lipid oxidation.

### 2.7. Statistical Analysis

All of the treatments were replicated three times, and the results were expressed as means ± standard deviations. The statistical analysis was performed in SPSS 20 software (SPSS Inc., Chicago, IL, USA). One-way analysis of variance (ANOVA) was used to analyze the statistical differences in the results, and Duncan’s multiple range test was used to distinguish significant differences between means. The significance level was set at 0.05. Linear regression analysis and curve prediction were performed using ORIGIN 2020b software.

## 3. Results and Discussion

### 3.1. Effect of Treatment Voltage on POV

**Figure 2:**
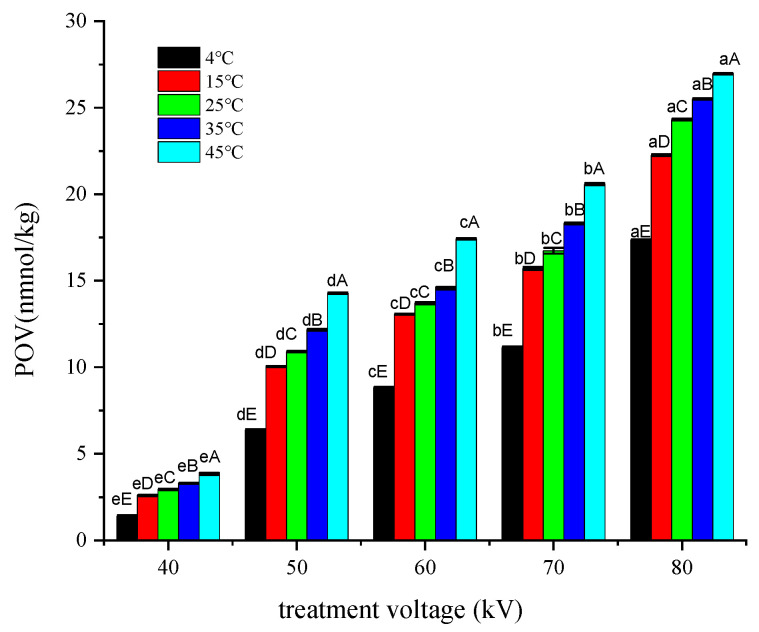
Changes in POV under different treatment voltages. Note: lowercase letters indicate differences among voltage treatment groups; capital letters indicate differences among storage temperature groups.

As shown in Figure 2, the POV of the fillets significantly increased (*p* < 0.05) with increasing voltage when the samples were stored under one temperature. If the samples were treated at the same voltage, increasing the storage temperature led to a significant increase in POV (*p* < 0.05). When the samples were treated at less than 70 kV and stored at 4 °C, the POV of tilapia fillets did not exceed the acceptable level limit (9.85 mmol/kg) [20]. Chen et al. [21] found no significant changes in the POV of *Scomber scombrus* treated at 60 kV for 2 min. However, Albertos et al. [11] treated *Scomber scombrus* with a DBD system, which resulted in a high POV, indicating that cold plasma treatment intensified the primary oxidation of lipids.

### 3.2. Effect of Treatment Time on POV 

**Figure 3:**
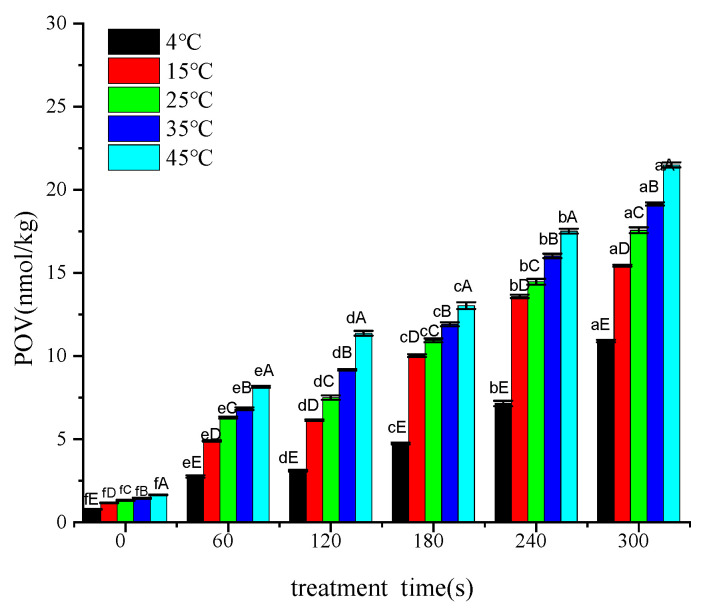
Changes in POV under different treatment times. Note: lowercase letters indicate differences among voltage treatment groups; capital letters indicate differences among storage temperature groups.

Increasing the treatment time of cold plasma and increasing the storage temperature both led to a deeper degree of primary lipid oxidation (Figure 3). When the storage temperature increased from 4 °C to 15 °C under the same treatment time, the POV increased the most, indicating that the primary oxidation of lipids was more rapid in the temperature range of 4–15 °C compared with the other temperature ranges. This may have been related to the optimal temperatures for the activity of lipoxidase and peroxidase.

Linear equations describing POV changes at different storage temperatures were obtained based on the data in Figure 2 and Figure 3 using the treatment voltage or time as the *x*-axis and the increased value of POV as the *y*-axis. The slope (*k*) and correlation coefficient (R^2^) of the linear equations are shown in Table 1.

In Table 1, k represents the rate of change in POV per unit increase in treatment voltage (1 kV) or per unit increase in treatment time (3.75 s) at different storage temperatures. Linear regression was performed with the data in Table 1, with storage temperature as the *x*-axis and the slope of the linear regression line at different temperatures as the *y*-axis, resulting in the following equations: for treatment voltage, y1 = 0.0037x + 0.3755 (R^2^ = 0.9042); for treatment time, y2 = 0.0452ln(x) + 0.0545 (R^2^ = 0.9919). These equations represent the effect of treatment voltage or time on the rate of change in POV. By comparing the y-values of the two equations with temperatures between 4 and 45 °C, the effects of treatment voltage or time on the rate of change in POV could be determined. If y1 − y2 > 0, it indicates that the promoting effect of treatment voltage on POV is greater than that of treatment time, and vice versa. The inequality y1 − y2 > 0 always holds for x > 0, as y1 − y2 = 0.08186x − ln(x) + 7.102. Under our study conditions, the changing rate of POV per unit increase in treatment voltage was between 0.367 and 0.526 nmol/kg, and the changing rate of POV per unit increase in treatment time was 0.117 and 0.230 nmol/kg. Therefore, the treatment voltage had a greater promoting effect on the primary oxidation of lipids than the treatment time during the cold plasma treatment.

### 3.3. Effect of Cold Plasma Treatment Voltages and Time on the Activation Energy of Primary Lipid Oxidation

By plotting ln(K) as the *y*-axis (where K = POV) and 1/T as the *x*-axis, the Arrhenius equation can be represented as a linear function with a slope of $-\frac{E}{R}$ As R is a constant, the activation energy (*Ea*) of lipid oxidation under different treatment voltage conditions can be compared through the slope of the line. By substituting the data from Figure 2, the following equations were obtained: 40 kV: y = −3.9922x + 1.3037 (R^2^ = 0.9537); 50 kV: y = −3.0317x + 2.5878 (R^2^ = 0.9131); 60 kV: y = −2.4803x + 2.782 (R^2^ = 0.9006); 70 kV: y = −2.3105x + 2.9709 (R^2^ = 0.9115); and 80 kV: y = −1.7539x + 3.279 (R^2^ = 0.9411).

From the line’s slope, the *Ea* for primary lipid oxidation under different treatment voltages could be obtained. At 40 kV: *Ea* = 33.19 kJ/mol; 50 kV: *Ea* = 25.21 kJ/mol, 60 kV: *Ea* = 20.62 kJ/mol; 70 kV: *Ea* = 19.21 kJ/mol; and at 80 kV: *Ea* = 14.58 kJ/mol. A linear equation was fitted with *Ea* on the *y*-axis and treatment voltage on the *x*-axis, yielding yA = −0.4321x + 48.491 (R^2^ = 0.9401), which represented the trend of *Ea* changes with treatment voltage. When the treatment voltage changed from 40 to 80 kV, the *Ea* changed from 14.58 to 33.19 kJ/mol.

Linear fitting was performed on the data in Figure 3 to obtain the linear equations for different treatment times. The equations obtained are as follows: 0 s: y = −2.8325x + 0.4369 (R^2^ = 0.9166); 60 s: y = −4.2392x + 2.0372 (R^2^ = 0.9245); 120 s: y = −4.94x + 2.3243 (R^2^ = 0.9044); 180 s: y = −4.2197x + 2.6032 (R^2^ = 0.9918); 240 s: y = −3.6854x + 2.8785 (R^2^ = 0.9829); and 300 s: y = −2.5897x + 3.0133 (R^2^ = 0.904). The *Ea* corresponding to the slopes were as follows: 0 s: *Ea* = 23.55 (kJ/mol), 60 s: *Ea* = 35.24 (kJ/mol), 120 s: *Ea* = 41.07 (kJ/mol), 180 s: *Ea* = 35.08 (kJ/mol), 240 s: *Ea* = 30.64 (kJ/mol), 300 s: *Ea* = 21.53 (kJ/mol). A curve function was obtained by fitting the change in *Ea* against the treatment time: yB = −0.0007x^2^ + 0.2017x + 24.685 (R^2^ = 0.9367). This indicated that the *Ea* change followed a quadratic function against the treatment time, with vertex coordinates of 140.4 and 39.21. When the treatment time changed from 0 to 300 s, the *Ea* changed from 21.53 to 41.07kJ/mol.

### 3.4. Effect of Treatment Voltage on TBARS Values

**Figure 4:**
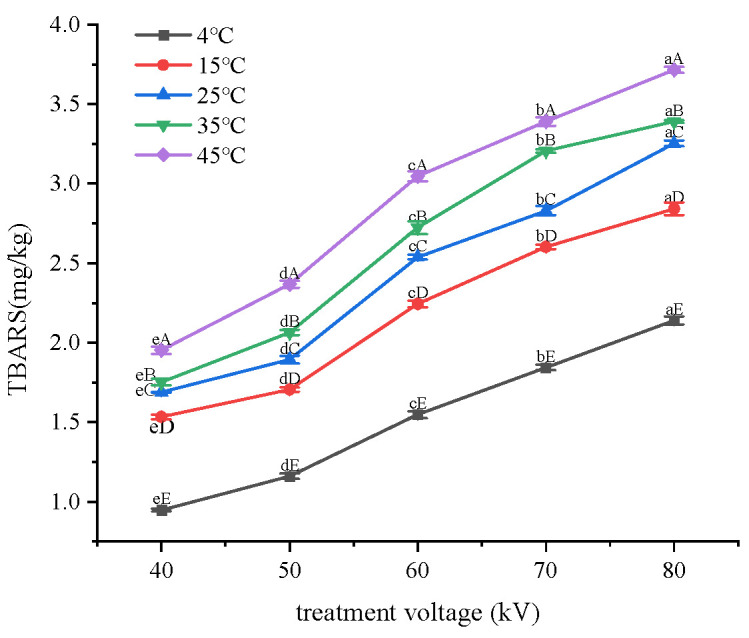
Changes in TBARS values under different treatment voltages. Note: lowercase letters indicate differences among voltage treatment groups; capital letters indicate differences among storage temperature groups.

As shown in Figure 4, increasing the treatment voltage significantly (*p* < 0.05) increased the TBARS values, and the rise in storage temperature further promoted the secondary oxidation of lipids. High contents of oxidation products of secondary lipid oxidation, such as aldehydes, alcohols, ketones, acids, and other small molecules, can lead to a deterioration in the taste of food samples [22]. In this study, the treatment and storage conditions of samples with acceptable TBARS are as follows: stored at 4 °C after 40–70 kV treatment; stored at 15 °C after 40–50 kV treatment; and stored at 25 °C after 40 kV treatment. The results indicate that multiple factors, including plasma treatment and external conditions, jointly affected the TBARS in fish flesh.

### 3.5. Effect of Treatment Time on TBARS Values

**Figure 5:**
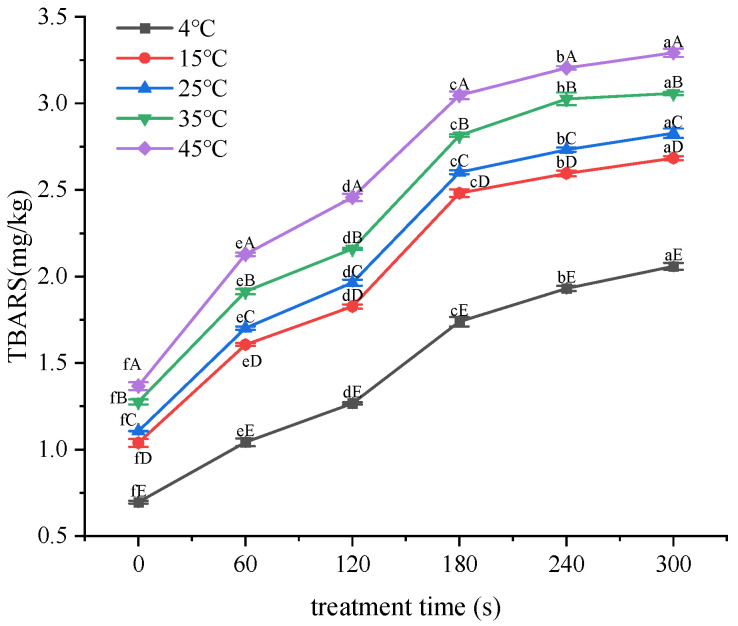
Changes in TBARS values under different treatment times. Note: lowercase letters indicate differences among voltage treatment groups; capital letters indicate differences among storage temperature groups.

As shown in Figure 5, the effect of treatment time on TBARS at different storage temperatures was similar to the effect of treatment voltage. The treatment and storage conditions of samples with acceptable TBARS were as follows: stored at 4 °C after 1–3 min treatment; stored at 15 °C after 1–2 min treatment; and stored at 25 °C after 1 min treatment.

Linear regression was performed on the data in Figure 4 and Figure 5, with voltage or treatment time as the *x*-axis and the increased value in TBARS as the *y*-axis. Linear equations for the changes in TBARS under different storage temperatures were obtained. The slope (k) and correlation coefficient (R^2^) of the linear equations are shown in Table 2.

Based on the fitting results above, a curve was obtained by fitting the slopes of the straight lines at different storage temperatures, with storage temperature as the *x*-axis and slope as the *y*-axis. For treatment voltage, y3 = 0.0004x + 0.0298 (R^2^ = 0.9636); for treatment time, y4 = 0.0001x+0.0183 (R^2^ = 0.9179). The fitting equations represent the effect of treatment voltage or treatment time on the rate of TBARS increase. The magnitude of the slopes of the two fitting lines reflected the promoting effect of the two factors on the rate of TBARS increase. As the slope of y3 was greater than that of y4, treatment voltage had a greater promoting effect on TBARS in tilapia fillets than treatment time. Therefore, increasing the voltage had a stronger promoting effect on secondary lipid oxidation than extending the treatment time.

### 3.6. Effect of Cold Plasma Treatment Voltages and Time on the Activation Energy of Secondary Lipid Oxidation

The data in Figure 4 were brought into the Arrhenius equation to obtain a linear equation for the change in TBARS for different voltages. The equations are as follows: 40 kV: y = ±2.9005x + 0.6625 (R^2^ = 0.9758); 50 kV: y = −2.6925x + 0.8011 (R^2^ = 0.9118); 60 kV: y = −2.6212x + 1.0722 (R^2^ = 0.9276); 70 kV: y = −2.4059x + 1.1951 (R^2^ = 0.9229); and 80 kV: y = −2.1454x + 1.2785 (R^2^ = 0.9161).

The *Ea* for secondary lipid oxidation corresponding to different treatment voltages could be determined by calculating the slopes of the equations provided above. The results were as follows: 40 kV: *Ea* = 24.11 kJ/mol, 50 kV: *Ea* = 22.39 kJ/mol, 60 kV: *Ea* = 21.79 kJ/mol, 70 kV: *Ea* = 20.00 kJ/mol, and 80 kV: *Ea* = 17.84 kJ/mol. The values of *Ea* for different processing voltages were fitted to obtain an equation: yC = −0.1494x + 30.1896 (R^2^ = 0.9607). When the treatment voltage changed from 40 to 80 kV, *Ea* changed from 17.84 to 24.11 kJ/mol.

The data in Figure 5 were brought into the Arrhenius equation to obtain a linear equation for the change in TBARS for different voltages. The equations were as follows: 0 s: y = −2.6544x + 0.2824 (R^2^ = 0.9287); 60 s: y = −2.7607x + 0.715 (R^2^ = 0.9353); 120 s: y = −2.4739x + 0.8363 (R^2^ = 0.9142); 180 s: y = −2.2857x + 1.1097 (R^2^ = 0.9154); 240 s: y = −1.9816x + 1.1392 (R^2^ = 0.9261); and 300 s: y = −1.7973x + 1.1579 (R^2^ = 0.9187).

The above equations yielded the slopes corresponding to the lipid secondary oxidation at different treatment times, resulting in the *Ea* for each interval. For 0 s: 22.07 kJ/mol; 60 s: 22.95 kJ/mol; 120 s: 20.57 kJ/mol; 180 s: 19.00 kJ/mol; 240 s: 16.48 kJ/mol; and for 300 s: 14.94 kJ/mol. By plotting the treatment time on the *x*-axis and corresponding *Ea* on the *y*-axis, a curve-fitting equation was derived: yD = −0.027x + 23.38 (R^2^ = 0.9224). When the treatment time changed from 0 to 300 s, *Ea* changed from 14.94 to 22.07 kJ/mol.

### 3.7. Effects of Treatment Voltages on Lipid Oxidation

**Figure 6:**
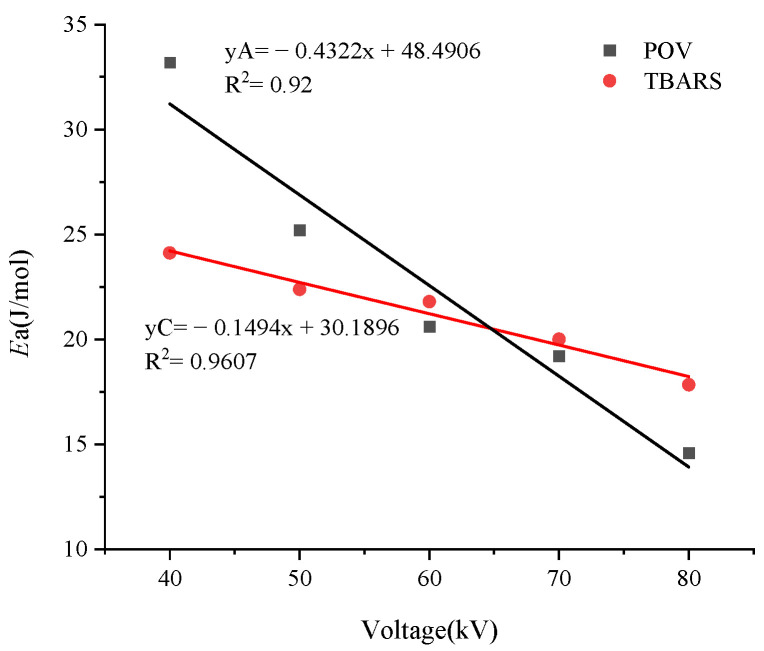
Changes in *Ea* for primary and secondary lipid oxidation under different voltages.

The linear equations of lipid primary and secondary oxidation of *Ea* were compared and analyzed in Figure 6. The intersection of the two lines was located at 64.71 kV, indicating that, if the treatment voltage was changed between 40 and 64.71 kV, the *Ea* required for primary lipid oxidation was higher than that for secondary oxidation; secondary lipid oxidation could easily occur after treatment within this range of voltages. When the voltage was higher than 64.71 kV, the activation energy required for primary oxidation became lower than that for secondary oxidation. This indicates that increasing the treatment voltage could promote primary lipid oxidation more easily than secondary lipid oxidation.

### 3.8. Effects of Treatment Time on Lipid Oxidation

**Figure 7:**
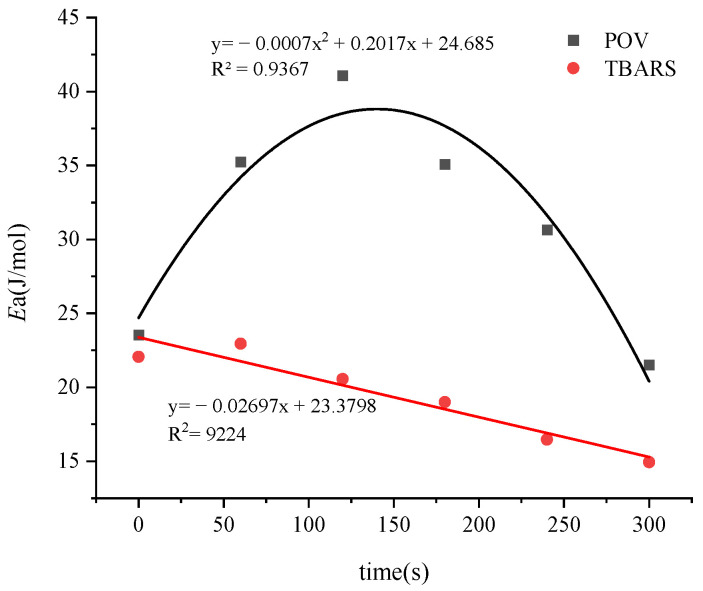
Changes in *Ea* for primary and secondary lipid oxidation under different processing times.

When the treatment time was increased from 0 to 300 s, the *Ea* for secondary lipid oxidation decreased as time increased, while the *Ea* for primary oxidation first increased and then decreased (Figure 7). Additionally, the *Ea* for secondary oxidation was lower than that for primary oxidation.

This suggests that, under the conditions in this study, extending the treatment time promoted the secondary oxidation of lipids.

Cold plasma has a good bactericidal effect for a variety of animal-derived foods and their products [23]. A large number of active free groups was formed during the treatment process, which caused the product quality to vary as the conditions of cold plasma and product types. Owing to the diversity of cold plasma treatment conditions and the obvious differences between various samples, studies have proven that cold plasma treatment can accelerate lipid oxidation. With the increase in treatment voltage and time of cold plasma, the POV of fresh mackerel (Scomber scombrus) slices increased significantly [11], while there was no significant difference in TBARS between the treatment and control groups, indicating that cold plasma treatment significantly promoted the primary oxidation process of fatty acids and had no significant effect on the secondary oxidation products of the samples. Albertos et al. [24] treated herring (Clupea harengus) with 70 kV cold plasma, and the TBARS of fish was higher than that of the untreated group after 9 d, indicating that the lipid oxidation secondary products gradually accumulated with the prolongation of storage time after treatment. Cold plasma treatment increased the TBARS value of dried black-billed fish, with changes in pH, color, and flavor [10]. The TBARS value of semi-dry saury [25] increased significantly after cold plasma treatment, but the POV did not change. The TBARS of dried squid increased with the extension of treatment time [26], which was proven to be a negative effect of treatment time on lipid oxidation once again. The same phenomenon was also found in salmon sushi [27] and smoked fish [28]. Above all, a long treatment time and high treatment voltage increased the primary and secondary oxidation of lipids. Therefore, to control the degree of lipid oxidation in samples treated by cold plasma, both treatment voltage and time should be considered together.

## 4. Conclusions

The treatment voltage and time of cold plasma treatment had significant effects on the POV and TBARS of tilapia fillets. The reaction rate constant was calculated and a well-correlated equation was fitted. The reaction rate constant was introduced to the Arrhenius equation to calculate the Ea of primary and secondary oxidation of lipids caused by the voltage and time factors, respectively. The results showed that the lipids were more prone to secondary oxidation when the voltage was between 40 and 64.71 kV; if the treatment voltage was higher than 64.71 kV, primary oxidation is more likely to occur; when the treatment time was in the range of 0–300 s, the Ea of primary oxidation increased first and then decreased, which was higher than that of secondary oxidation, indicating that the primary oxidation reaction was inhibited. Therefore, when choosing the conditions for plasma treatment, the degree of lipid oxidation of the samples should be taken into account to control the treatment time and voltage.

## Figures and Tables

**Table 1:** The slopes and correlation coefficients of the linear equations fitted by treatment voltage and time to POV.

	°C	4	15	25	35	45
Voltage	k	0.3668	0.4502	0.4856	0.5056	0.5257
	R^2^	0.9673	0.9654	0.9613	0.9603	0.9451
Time	k	0.1169	0.1809	0.1949	0.213	0.2304
	R^2^	0.9276	0.9865	0.9855	0.9880	0.9750

**Table 2:** The slopes and correlations coefficient of the linear equations fitted by voltage and time to TBARS.

	°C	4	15	25	35	45
Voltage	k	0.0307	0.0351	0.0407	0.0442	0.0455
	R^2^	0.9944	0.9769	0.9803	0.9716	0.9814
Time	k	0.0181	0.0214	0.0223	0.0233	0.0244
	R^2^	0.9684	0.9266	0.9343	0.9256	0.9174

## Data Availability

Data is contained within the article.
